# Neurocognitive, Autonomic, and Mood Effects of Adderall: A Pilot Study of Healthy College Students

**DOI:** 10.3390/pharmacy6030058

**Published:** 2018-06-27

**Authors:** Lisa L. Weyandt, Tara L. White, Bergljot Gyda Gudmundsdottir, Adam Z. Nitenson, Emma S. Rathkey, Kelvin A. De Leon, Stephanie A. Bjorn

**Affiliations:** 1 Department of Psychology, George and Anne Ryan Institute for Neuroscience, University of Rhode Island, Kingston, RI 02881, USA; 2 Center for Alcohol and Addiction Studies, Brown University, Providence, RI 02912, USA; Tara_White@Brown.Edu; 3 Department of Behavioral and Social Sciences, School of Public Health, Brown University, Providence, RI 02912, USA; 4 Carney Institute for Brain Science, Brown University, Providence, RI 02912, USA; 5 Breidholt Service Center, 109 Reykjavik, Iceland; gydagudmundsdottir@gmail.com; 6 Neuroscience Graduate Program, Brown University, Providence, RI 02912, USA; adam_nitenson@brown.edu (A.Z.N.); kelvin_deleon@brown.edu (K.A.D.L.); 7 School Psychology Graduate Program, University of Rhode Island, Kingston, RI 02881, USA; emma_rathkey@my.uri.edu; 8 Psychology Undergraduate Program, University of Rhode Island, Kingston, RI 02881, USA; stephanie_bjorn@my.uri.edu

**Keywords:** prescription stimulants, Adderall, neurocognitive enhancement, college students, prescription stimulant misuse

## Abstract

Prescription stimulant medications are considered a safe and long-term effective treatment for Attention Deficit Hyperactivity Disorder (ADHD). Studies support that stimulants enhance attention, memory, self-regulation and executive function in individuals with ADHD. Recent research, however, has found that many college students without ADHD report misusing prescription stimulants, primarily to enhance their cognitive abilities. This practice raises the question whether stimulants actually enhance cognitive functioning in college students without ADHD. We investigated the effects of mixed-salts amphetamine (i.e., Adderall, 30 mg) on cognitive, autonomic and emotional functioning in a pilot sample of healthy college students without ADHD (*n* = 13), using a double-blind, placebo-controlled, within-subjects design. The present study was the first to explore cognitive effects in conjunction with mood, autonomic effects, and self-perceptions of cognitive enhancement. Results revealed that Adderall had minimal, but mixed, effects on cognitive processes relevant to neurocognitive enhancement (small effects), and substantial effects on autonomic responses, subjective drug experiences, and positive states of activated emotion (large effects). Overall, the present findings indicate dissociation between the effects of Adderall on activation and neurocognition, and more importantly, contrary to common belief, Adderall had little impact on neurocognitive performance in healthy college students. Given the pilot design of the study and small sample size these findings should be interpreted cautiously. The results have implications for future studies and the education of healthy college students and adults who commonly use Adderall to enhance neurocognition.

## 1. Introduction

Attention-deficit/hyperactivity disorder (ADHD) is a chronic disorder characterized by developmentally inappropriate levels of inattention and/or hyperactivity-impulsivity that affects approximately 3–10% of school age children and adolescents [1]. Research indicates that increasing numbers of high school students with ADHD are pursuing college and in a recent national survey, 6.4% of male and 3.9% of female freshman reported having ADHD [2]. College students with ADHD are entitled by the American Disabilities Act to receive educational support services, and approximately 25% of college students receiving disability support services receive services for ADHD. This percentage has increased substantially since 1975 [3]. The prognosis for college students with ADHD varies across studies, but most suggest that college students with ADHD are at increased risk for academic problems, executive function deficits, lower grade point average, compromised academic coping skills, and higher than average risk for psychological and interpersonal difficulties [4,5,6,7,8,9]. A number of non-pharmacological treatments are available for ADHD (e.g., behavioral support therapies, cognitive-behavior therapy, biofeedback) and studies attest to their varying effectiveness. To date, however, the majority of ADHD treatment outcome studies have shown that, in general, the most effective treatment for reducing ADHD symptoms is stimulant medication (e.g., amphetamine, methylphenidate) although questions remain regarding the magnitude of these effects given various methodological problems in the literature [10]. Questions also remain regarding the extent to which placebo effects and responding contributes to observed improvements in cognitive and behavioral functioning in children and adults. For example, Fageera and colleagues [11] recently found a significant placebo response in both parents and teachers of children with ADHD during a randomized, placebo controlled clinical trial of methylphenidate with more robust effects observed in parents than teachers. Mateeton et al. [12] reported that lisdexamfetamine (LDX) is efficacious and well tolerated in the treatment of ADHD in adults, however its acceptability is no higher than placebo. With respect to college students, Weyandt and colleagues [13] were the first to demonstrate the effectiveness and safety of lisdexamfetmaine dimesylate (LDX; Vyvanse) in college students with ADHD in the context of a double-blind, placebo-controlled crossover study. Specifically, DuPaul and colleagues [14] and Weyandt et al. [13] found that lisdexamfetmaine dimesylate was effective at reducing ADHD symptomatology and at improving cognitive skills, including executive functions, in college students with documented ADHD. 

Although prescription stimulants are often highly effective in reducing ADHD symptoms, non-medical use of prescription stimulants among college students without the disorder has become increasingly problematic in recent years. Non-medical use, or misuse, of stimulants has been defined as taking stimulants without a valid prescription and/or use of stimulants other than as prescribed [13,15]. Over a decade ago, Babcock and Byrne [16] surveyed 283 students regarding misuse of prescription stimulants and found that 16% of the sample reported taking Ritalin for “fun”; yet, less than 2% of the sample had a current prescription for Ritalin. McCabe, Knight, Teter, and Wechsler [17] found that 6.9% of college students surveyed reported lifetime misuse of prescription stimulants. Further, DuPont et al. [18] found the most frequently reported motive for misuse of stimulant medication was to “work/study” and to “party.” Similarly, Weyandt et al. [19] Judson and Langdon [20], and Bossaer et al. [21] found that college students who misused prescription stimulants did so to enhance cognitive functioning, such as concentration, and to increase alertness or to stay awake. In a systematic review of 22 studies, Weyandt et al. [13] found that 5–35% of college students surveyed had misused prescription stimulants primarily for neurocognitive enhancement. Other reasons commonly reported by students include recreational use (e.g., partying, pharming, weight loss) and some students (40%) appear to misuse prescription stimulants for both cognitive enhancement and recreational purposes [15]. Munro, Weyandt, Marraccini, and Oster [22] recently studied college students from six public universities located in various regions of the United States and reported that students with clinically significant executive function deficits reported significantly higher rates of prescription stimulant misuse. Prescription stimulant misuse has also been reported among college students in other countries including Germany [23], Iceland [24], Switzerland [25], and the United Kingdom [26] with rates similar to those found in the USA. Interestingly, and similar to results from studies conducted in the USA, Gudmundsdottir and colleagues [24] found that ADHD symptomatology and anxiety was significantly associated with prescription stimulant misuse behavior. Other international studies have reported similar findings [27] underscoring that this behavior is present across cultures.

Contrary to student expectations and beliefs, prescription stimulants may not lead to neurocognitive enhancement and improved academic performance [15], as stimulant misuse has been found to be negatively correlated with academic functioning [13,28]. Weyandt and colleagues [13] emphasized that empirical studies are needed to determine whether stimulants are truly neurocognitive enhancers for college students without ADHD. Ilieva, Boland, and Farah [29] explored both the objective and subjective effects of amphetamines on cognition among healthy adults and did not find support for significant effects of amphetamine on episodic memory, working memory, inhibitory control, creativity, intelligence and scholastic achievement. Participants, however, did report perceived neurocognitive enhancement suggesting a placebo effect likely accounted for the perceived cognitive enhancement. In contrast, Smith and Farah [30] conducted a review of studies investigating neurocognitive effects of prescription stimulants in healthy adults and reported that both amphetamine and methylphenidate were associated with overall improvements (accuracy and/or speeded responses) in response inhibition tasks as well as perceptual motor and delay rewards tasks. Interestingly, however, stimulants were found to impair performance on tasks of response inhibition requiring cancellation (i.e., stop signal tasks), cognitive flexibility (i.e., Intra-Extra Dimensional Set-shift Task, Wisconsin Card Sorting Task), trail-making tasks and reversal learning tasks. Amphetamine can induce reductions and increases in incentive-related risk behavior, depending on the individual being tested [31]. As Marraccini, Weyandt, Rossi, and Gudmundsdottir [32] (noted, these findings suggest that baseline performance is a crucial moderator and depending on baseline levels, stimulants may result in improvement or worsening of cognitive functioning and those with lower baseline functioning may derive the greatest benefits. Other studies, however, have reported mixed findings with healthy adults, with some supporting positive and large effects of methylphenidate on both immediate and delayed working memory and episodic memory [33] as well as processing speed [32]. In summary, some preliminary evidence suggests that healthy adults without ADHD may receive small to moderate cognitive benefits from prescription stimulant medication (e.g., Adderall) in the areas of working memory, response inhibition, processing speed, and delayed memory; however, questions remain regarding the effects of prescription stimulants on measures of attention, executive function (e.g., decision making, verbal fluency, and planning) and other aspects of cognition. A dearth of information exists regarding the potential effects of Adderall on mood and self-perceived cognitive enhancement effects of Adderall. 

The acute neurocognitive effects of Adderall will be best understood in the larger context of the drug’s concurrent effects on emotion, subjective state, and self-perceived cognitive ability. These domains are readily evaluated by self-report measures such as the Drug Effects Questionnaire, which evaluates subjective ratings of drug liking and drug high [34]; the Positive Activation scale, which evaluates the presence and intensity of activated emotion such as elation and euphoria [35]; and the Perceived Drug Effect Self-Report scale, a measure of that evaluates personal perceptions of whether one’s cognitive ability has improved after drug consumption [29]. Psychostimulant-induced changes in activated positive emotion, subjective drug responses, and autonomic activation are typically large in size [36,37,38], with a smaller evidentiary base regarding psychostimulant drug effects on self-perceptions of cognitive functioning [29]. Very few studies evaluate Adderall effects on neurocognitive function in the context of drug-induced changes in activated emotion, subjective drug experience, autonomic activation, and changes in meta-cognition. Joint assessment of Adderall effects on neurocognition, mood, activation, and perceived cognitive enhancement would thus provide important and highly novel information through which to better understand the potential effects of Adderall on neurocognition as opposed to other outcomes that may affect drug choice behavior. 

Given that misuse of prescription stimulants is widely reported on college campuses for the purpose of neurocognitive enhancement, and that students typically view these drugs as safe, research is sorely needed to empirically address the joint neurocognitive and meta-cognitive activational effects of prescription stimulants in college students without ADHD. Therefore, the purpose of the present pilot study was to explore: (a) the potential effects of the prescription stimulant Adderall on the cognitive performance of healthy college students without ADHD in the areas of memory, reading comprehension, sustained attention, impulsivity, and executive function relative to placebo; and (b) concomitant effects of Adderall on autonomic activation, subjective drug responses and activated emotion in healthy college students without ADHD. This approach provides an empirical test of Adderall effects on cognitive function, emotion and autonomic activity, and concurrent estimates of drug impact on each outcome. We hypothesized that participant performance on measures of cognition would be significantly enhanced after ingestion of Adderall compared to placebo. Specifically, we expected enhanced performance after Adderall (30 mg) compared to placebo for (1) reading comprehension; (2) working memory; (3) executive function; and (4) greater self-perceptions of performance on cognitive tasks. Understanding potential effects of stimulants on these outcomes is important for prevention efforts and mitigating prescription drug misuse among healthy college students.

## 2. Methods

### Study Design

**Figure 1:**
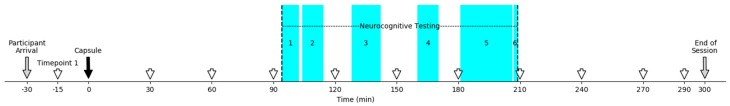
Experimental Design. Legend: Timing of test sessions. Sessions were 5.5 h in duration (330 min. total). X-axis denotes time relative to administration of the blinded study capsule at time 0 (black arrow). Neurocognitive testing began approximately 90 min after administration of the study capsule (vertical dashed black line). Six neurocognitive tests (Tasks 1–6; with tasks 1–5 randomized for order across participants; see Methods section for details) were conducted over a two-hour time period between 90 and 210 min post-capsule (blue shading). An example order (Tasks 1–6) is presented to illustrate timing of assessment. Autonomic and self-reported subjective states were evaluated at half hour intervals, for eleven (11) timepoints of assessment per test session (open arrows). Participant arrival and departure are indicated (gray arrows). Each participant completed two test sessions (Placebo and Adderall). Order of drug administration was counterbalanced across participants, using a crossover design.

This pilot study employed a double-blind, placebo-controlled, within-subjects crossover design. Cognitive tasks were administered during the peak period of the drug effect, between 90 and 210 min after the capsule was administered. The study design is illustrated in Figure 1. 

## 3. Procedure

The study was approved by the Institution Review Board for research with human subjects at the University of Rhode Island, Brown University, and the Memorial Hospital of Rhode Island in accordance with the Code of Federal Regulations (Title 45, Part 46) “Protection of Human Subjects” adopted by the National Institutes of Health and the Office for Protection from Research Risks. The study was conducted ethically in accordance with the Helsinki Declaration of 1964 (revised 2013) and the National Advisory Council on Drug Abuse Recommended Guidelines for the Administration of Drugs to Human Subjects.

Healthy college student participants (i.e., college students without ADHD or other psychiatric conditions) were recruited via campus flyers, announcements in courses, and advertisements. Interested students contacted the researchers via email or telephone and completed a telephone screening to determine initial eligibility, followed by an in-person session in which written informed consent was obtained. The Mini-International Neuropsychiatric Interview (M.I.N.I. (Version 5.0.0); [39]) was administered to exclude participants meeting criteria for other psychiatric disorders from the sample. Participants were then scheduled for a medical exam and electrocardiogram (EKG) at a local hospital. To be included in the study, participants had to be between the ages of 18 and 24, physically healthy, have a normal EKG, and not be taking any prescription medication for a chronic medical condition in the past 6 months with the exclusion of antibiotics, or birth control pills in female participants. Once cleared for the study, participants took part in two test sessions in which a capsule was administered and neurocognitive, physiological and mood assessments were conducted. Test sessions used a time-locked protocol that lasted 5.5 h apiece, with test sessions scheduled at the same time of day to eliminate circadian effects. The order of placebo and active drug was counterbalanced for order across participants. On each test day, participants arrived at the lab and completed a brief pre-protocol readiness assessment, and baseline self-report and physiological measures (heart rate, diastolic and systolic blood pressure measures). Thirty minutes after arrival, participants received an opaque capsule that contained the active drug or placebo, which was consumed with a glass of water and verified by visual inspection of the oral cavity. Amphetamine mixed salts (i.e., generic Adderall) and placebo (dextrose) were selected as the study drugs because Adderall and its generic equivalent is an FDA-approved treatment for ADHD that is prone to misuse in college populations, has a known safety profile and is one of the most commonly prescribed psychostimulants for adults with ADHD (MTA Group, 1999). The 30 mg oral dose was selected because it produces significant effects on attention in the context of ADHD, and has a well-documented safety profile in young adults [40]. Mixed amphetamine salts (30 mg oral) were compounded with dextrose filler and dispensed as individual prescriptions in opaque, colored gelatin capsules. The placebo consisted of identical gelatin capsules and contained only dextrose. The dosing schedule followed a standardized period of fasting (2 h), with neurocognitive assessment over a two-hour period 90–210 min post-consumption to ensure that cognitive assessments occurred during the peak period of pharmacological effects. Autonomic and mood assessment was conducted every 30 min (Figure 1).

Across the study, 53 college students were contacted who expressed interest in the study, 21 of who were ineligible, typically due to recreational drug use and medical conditions/medication. Seven individuals opted not to continue with the study, and one individual declined the study screening. This resulted in 24 in-person interview sessions where participants were informed of the nature of the study, provided written consent, and enrolled in the study. Four participants were excluded after enrollment, and seven dropped out post-consent due to scheduling constraints, lack of continued interest, or new medical issues not present at initial screening. The remaining 13 participants completed the study in full, yielding 26 sessions of data collection for analysis.

## 4. Instruments

### Neurocognitive Measures

For each participant, the six tasks described below were administered randomly, to reduce potential order effects. For each participant, the order of tasks was recorded on the first session, and the same sequence was administered on their second test session. For all participants, Task 2 and Task 5 were never administered sequentially as they each measure aspects of oral language, and Task 6 was always presented last. An example order of task administration is presented in Figure 1. Tasks are described below.

Task 1. Digit Span Forward and Backward [41]. This task evaluates working memory and is derived from a subtest of the Wechsler Adult Intelligence Scale, fourth edition [41] For Digit Span Forward, the participant was read a sequence of numbers and was asked to verbally recall the numbers in the same order. For Digit Span Backward, the examinee was read a sequence of numbers and was asked to verbally recall the numbers in reverse order. Digit Span measures working memory, mental manipulation, cognitive flexibility, rote memory and learning, attention, and encoding. The primary dependent variable was the total age normed scaled score for digit span forward and backward. Total time to complete the Digit Span tasks was approximately 8 min.

Task 2. Story Recall subtest from the Woodcock Johnson–III [42]. This task measures aspects of oral language including language development and meaningful memory. The task required the participants to recall increasingly complex stories. After listening to a passage, the participant was asked to recall as many details of the story as they could remember. All participants began the task according to their age range as predetermined by the test’s standardized procedures. The dependent measure on the task was the WJ total age normed scale score. Total time to complete the task was 10 min.

Task 3. Conners Continuous Performance Test Third Edition [43]. The CPT 3 was administered to measure Inattentiveness, Impulsivity, Sustained Attention, and Vigilance. The administration of the CPT 3 involves 360 trials and requires respondents to press the spacebar whenever any letter, except the letter “X”, appears on the computer screen. Measures of Inattentiveness include lower Detectability (d’), a larger number of Omissions and Commissions, a slower Hit Reaction Time (HRT), as well as greater inconsistencies in response speed (HRT Standard Deviation and Variability). On the other hand, a faster HRT and a larger number of Commissions and/or Perseverations serve as a measure of Impulsivity. T-scores for HRT, errors of Omission and Commission and Variability were the dependent variables. Time to complete the CPT 3 was 14 min.

Task 4. The Behavior Rating Inventory of Executive Function, Adult version (BRIEF-A; [44]). This standardized, norm referenced, self-report instrument measures executive functions and self-regulation abilities in daily living. The three dependent variables from the BRIEF-A were total scores from the Behavioral Regulation Index (BRI), Metacognition Index (MI), and Global Executive Composite (GEC). The BRIEF-A was included to gather information regarding the potential drug effect of Adderall on participants memory of past cognition and self-regulation ability. Time to complete task was approximately 10 min.

Task 5. Gray Oral Reading Tests−Fifth Edition (GORT-5; [45]). This task measures oral reading rate, accuracy, fluency, and comprehension. There are two test forms, each containing 14 reading sequences and relevant questions. The dependent variable was the Oral Reading Index scaled score, which provides a combined measure of reading fluency and comprehension. Time to complete the task was approximately 20–30 min.

Task 6. Perceived Drug Effect Self-Report (PDE-SR) [29] This is a one-item measure of whether participants perceived the drug as influencing their performance on neurocognitive tasks and was administered at the end of neurocognitive testing. The measure reads: ‘The following question refers to all tests completed TODAY. How and how much did the drug influence (either positively or negatively) your performance on the tests? Please use the scale below. Your answer can be any number between 1 and 100.” The scale was a line, ranging from 1 to 100, labeled as follows: 1 = “drug impaired my performance extremely”; 25 = “the drug somewhat impaired my performance”; 50 = “the drug had no effect”; 75 = “the drug somewhat improved my performance”; 100 = “the drug improved my performance extremely.” The dependent variable was the self-reported score of the perceived drug effect on neurocognitive performance.

## 5. Activational Measures

Subjective Drug Effects. Drug Effects Questionnaire (DEQ; [34]). The DEQ consists of four questions concerning subjective drug effects. Participants indicate on 100-mm lines their response to four items: (1) ‘I FEEL some drug effects right now’, rated from “not at all” to “a lot”; (DEQ-Feel Drug); (2) ‘I LIKE the effects I am feeling right now’, rated from “dislike” to “like very much” (DEQ-Like); (3) ‘I am HIGH’, rated from “not at all” to “very much” (DEQ-High); and (4) ‘I would like MORE of what I consumed right now’, rated from “not at all” to “very much” (DEQ-Want More). Prescription psychostimulants such as d-amphetamine produce robust increases on these measures in healthy young adults [36,46,47]. DEQ measures were administered at half hour intervals, for a total of 11 timepoints of assessment on each session (details in Figure 1).

Activated Emotion. Positive Activation [35]. Positive Activation (PA) consists of 10 ratings of activated positive emotion. Participants circle a response on the PA scale from 0 to 9, ranging from the relative absence (0) to the strong presence (9) of positively valenced activated emotion (0 = depressed/sluggish; 1 = dull/tired; 2 = pleasant/fresh; 3 = cheerful/lively; 4 = delighted/energetic’; 5 = enthused/peppy; 6 = thrilled/strong; 7 = exuberant/vigorous; 8 = elated/exhilarated; 9 = ecstatic/invincible). A parallel scale of Negative Activation (NA), developed at the same time by our group [35], which consists of 10 ratings of activated negative emotion, was evaluated to provide information on specificity and discriminant validity. Participants circle a response on the NA scale from 0 to 9, ranging from the relative absence (0) to the strong presence (9) of negatively valenced activated emotion (0 = placid; 1 = calm; 2 = relaxed; 3 = annoyed; 4 = tense; 5 = nervous; 6 = distressed; 7 = jittery; 8 = hostile; 9 = contemptful). Dopamine agonists and incentive stimuli produce increases in PA in healthy volunteers [35,37,47,48]. PA and NA were evaluated by each participant at half hour intervals throughout the protocol (11 timepoints per session; Figure 1).

Autonomic Activation. Heart rate, diastolic blood pressure and systolic blood pressure were assessed at half hour intervals (11 time points per session; Figure 1). Prescription psychostimulants produce robust increases in autonomic activity in young adults [36,49]. The autonomic measures provide substantiating evidence of drug exposure and drug efficacy during the protocol.

## 6. Data Analyses

The neurocognitive hypotheses—that participant performance on measures of neurocognition would be significantly better after ingestion of Adderall compared to placebo—were tested by paired-samples *t*-tests (1-tailed). Cohen’s *d* effect sizes were computed to ascertain effect size estimates for Adderall effects on neurocognitive performance, with Cohen’s *d* values between 0.2 and 0.5 considered small effects, 0.5 and 0.8 considered medium effects, and above 0.8 considered large effects [50].

Activational hypotheses—that subjective effects, activated emotion and autonomic activity would be significantly higher after Adderall than placebo—were tested by within-subjects, repeated-measures drug (PBO, Adderall) by time (11 time points) 2-way ANOVA. For drug main effects, partial eta-squared values were calculated to ascertain effect size estimates of Adderall effects on activation across the 5.5 h testing period, with partial eta-squared values of 0.01 considered small effects, 0.06 considered medium effects, and 0.14 considered large effects [50,51,52] Cohen’s *d* effect sizes were computed to ascertain effect size estimates for Adderall effects on activation at the time point of maximal drug response, with Cohen’s *d* values between 0.2 and 0.5 considered small effects, 0.5 and 0.8 considered medium effects, and above 0.8 considered large effects [50].

## 7. Statistical Power

Power analyses for within-subjects effects were conducted in G*Power 3.1 using an alpha of 0.05 [51,53]. The final sample of 13 had high power (1 − β = 0.86) to detect large drug effects (*d* = 0.80), moderate power (1 − β = 0.52) to detect medium drug effects (*d* ≥ 0.50), and low power (1 − β = 0.17) to detect small drug effects (*d* = 0.20). 

## 8. Results

### 8.1. Descriptive Statistics

Table 1 depicts the means and standard deviations for neurocognitive performance measures during the Adderall and placebo sessions. Adderall had minimal, but mixed, effects on cognitive processes relevant to neurocognitive enhancement. Drug effects were medium in size on CPT 3—Variability and Digit Span Forward, and small in size on BRIEF-A—BRI, CPT 3 Commission Errors, CPT 3 Hit Reaction Time, GORT—ORI, and Perceived Drug Effect Self-Report.

### 8.2. Drug Effects on Activation

Adderall had substantial effects on subjective drug experience, activated emotion and autonomic activity (average partial eta-squared = 0.40, range 0.31–0.52, indicating large effects across the session; and average Cohen’s *d* = 1.03, range 0.71–1.26, indicating medium to very large effects at the peak time point; see Table 2). These effects are described in turn.

**Figure 2:**
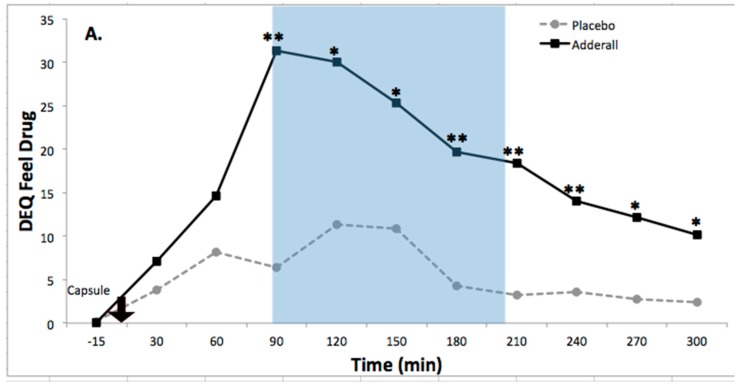

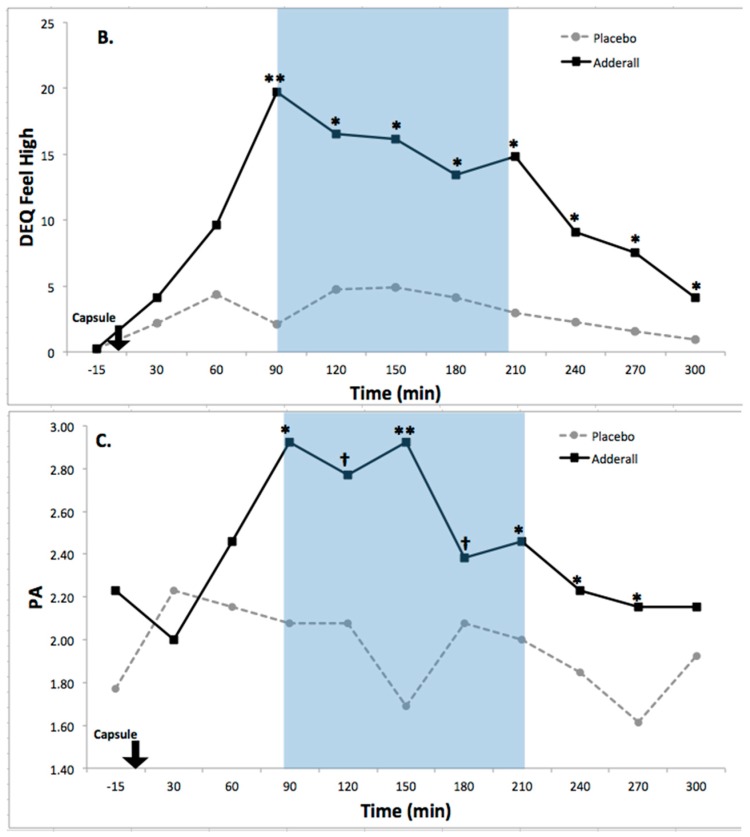
Effects of Adderall on Subjective State and Activated Emotion. Legend. (**A**) Adderall increased ratings of DEQ Feel Drug (Drug × Time Interaction, *F*(10,120) = 2.82, *p* < 0.005; Drug Main Effect, *F*(1,12) = 9.64, *p* < 0.01), a large effect (partial eta squared = 0.45; Cohen’s *d* = 1.26); (**B**) Adderall increased ratings of DEQ Feel High (Drug × Time Interaction, *F*(10,120) = 2.46, *p* ≤ 0.01; Drug Main Effect, *F*(1,12) = 7.06, *p* < 0.05), a large effect (partial eta squared = 0.37; Cohen’s *d* = 1.04); (**C**) Adderall increased ratings of Positive Activation (PA) (Drug × Time Interaction, *F*(10,120) = 2.20, *p* < 0.05; Drug Main Effect, *F*(1,12) = 5.27, *p* < 0.05), a large effect (partial eta squared = 0.31; Cohen’s *d* = 0.71). For all graphs, X-axis denotes time relative to administration of the blinded study capsule at time 0 (black arrow). DEQ = Drug Effects Questionnaire. Asterisks indicate differences between Adderall and Placebo, * *p* ≤ 0.05; ** *p* ≤ 0.01. Dagger symbols indicate trend-level differences, † *p* ≤ 0.10. Blue shading indicates time period of neurocognitive testing with tasks randomized per participant (see methods section for details). *N* = 13 participants in each drug condition, 26 sessions.

Subjective Drug Effects. Adderall significantly increased ratings of DEQ Feel Drug and DEQ Feel High compared to placebo (Table 2). For DEQ Feel Drug, there was a significant main effect of drug: *F*(1,12) = 9.64, *p* = 0.009 and a significant drug by time interaction: *F*(10,120) = 2.82, *p* = 0.004. These effect are illustrated in Figure 2A and were large in size, partial eta-squared = 0.45; Cohen’s *d* = 1.26. For DEQ Feel High, there was a significant main effect of drug: *F*(1,12) = 7.06, *p* = 0.02, and a significant drug by time interaction: *F*(10,120) = 2.46, *p* = 0.01. These effects are illustrated in Figure 2B were large in size, partial eta-squared = 0.37, Cohen’s *d* = 1.04.

Activated Emotion. Adderall significantly increased ratings of activated positive emotion (PA) compared to placebo (see Table 2). For PA, there was a significant main effect of drug: *F*(1,12) = 5.27, *p* = 0.04, and a significant drug by time interaction: *F*(10,120) = 2.20, *p* = 0.02. Drug effects on PA are illustrated in Figure 2C and were large in size across the session, partial eta-squared = 0.31, with medium effects at the peak time point, Cohen’s *d* = 0.71. There was no drug effect on negative emotion (NA), *p* > 0.44, n.s. These findings provide evidence of specificity and discriminant validity, and indicate a selective effect of Adderall on positively valenced states of activated emotion following drug consumption. 

**Figure 3:**
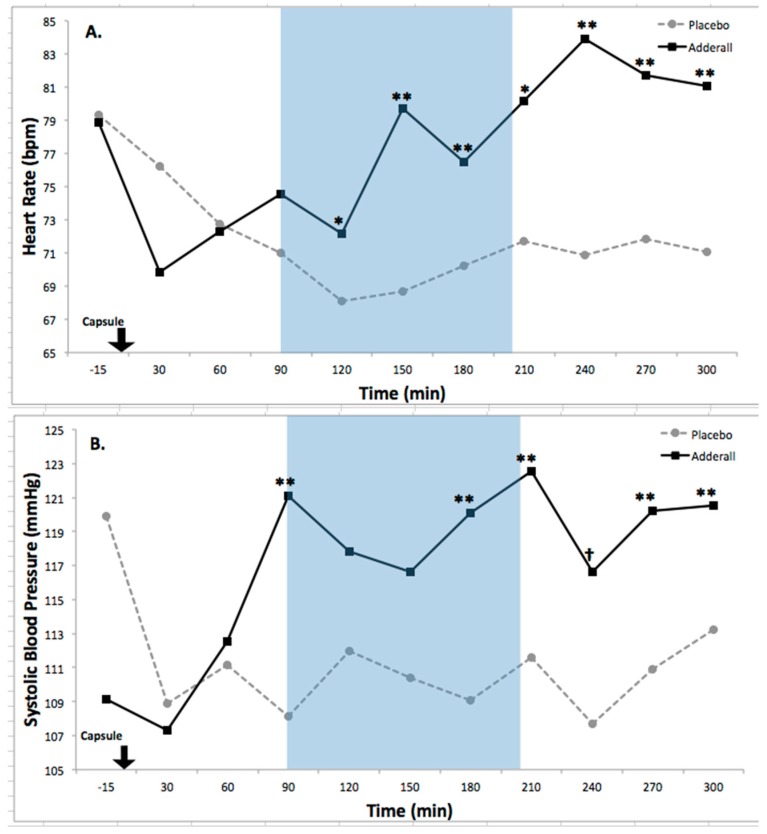

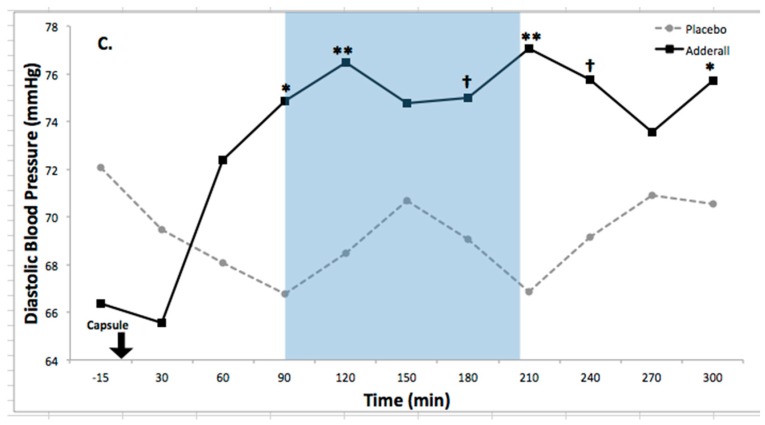
Effects of Adderall on Autonomic Activity. Legend. (**A**) Adderall increased Heart Rate (Drug × Time Interaction, *F*(10,120) = 4.30, *p* < 0.0001; Drug Main Effect, *F*(1,12) = 12.84, *p* < 0.005), a large effect (partial eta squared = 0.52, Cohen’s *d* = 1.25). (bpm) = beats per minute; (**B**) Adderall increased Systolic Blood Pressure (Drug × Time Interaction, *F*(10,120) = 3.36, *p* = 0.001; Drug Main Effect, *F*(1,12) = 10.1, *p* < 0.01), a large effect (partial eta squared = 0.46, Cohen’s *d* = 1.05); (**C**) Adderall increased Diastolic Blood Pressure (Drug × Time Interaction, *F*(10,120) = 3.28, *p* = 0.001; Drug Main Effect, *F*(1,12) = 5.38, *p* < 0.05), a large effect (partial eta squared = 0.31, Cohen’s *d* = 0.86). For all graphs, X-axis denotes time relative to administration of the blinded study capsule at time 0 (black arrow). Asterisks indicate differences between Adderall and Placebo, * *p* ≤ 0.05; ** *p* ≤ 0.01. Dagger symbols indicate trend-level differences, † *p* ≤ 0.10. Blue shading indicates time period of neurocognitive testing with tasks randomized per participant (see methods section for details). *N* = 13 participants in each drug condition, 26 sessions.

Autonomic Activity. Adderall significantly increased autonomic activity compared to placebo (Table 2). For heart rate there was a significant main effect of drug: *F*(1,12) = 12.84, *p* = 0.004 and a significant drug by time interaction: *F*(10,120) = 4.30, *p* = 0.00003. Drug effects on heart rate are illustrated in Figure 3A and were large in size, partial eta-squared = 0.52, Cohen’s *d* = 1.25. For systolic blood pressure there was a significant main effect of drug: *F*(1,12) = 10.1, *p* = 0.008, and a significant drug by time interaction: *F*(10,120) = 3.36, *p* = 0.001. These effects are illustrated in Figure 3B and were large in size, partial eta-squared = 0.46, Cohen’s *d* = 1.05. For diastolic blood pressure there was a significant main effect of drug: *F*(1,12) = 5.38, *p* = 0.04, and a significant drug by time interaction: *F*(10,120) = 3.28, *p* = 0.001. Effects on diastolic blood pressure are illustrated in Figure 3C and were large in size, partial eta-squared = 0.31, Cohen’s *d* = 0.86. These findings substantiate sympathomimetic drug activity during the protocol, providing evidence of internal validity of drug exposure (Table 2; Figure 3A–C). 

### 8.3. Drug Effects on Neurocognitive Functioning

**Figure 4:**
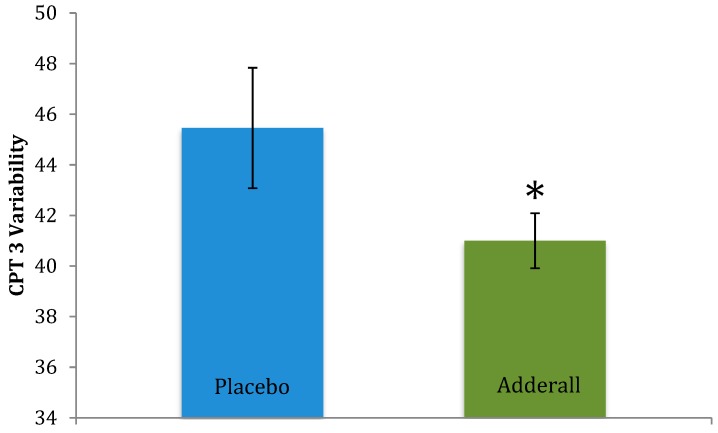
Effects of Adderall on CPT-3 Variability. Legend: Adderall significantly improved performance (i.e., reduced Variability): *t*(10) = −2.25, * *p* < 0.05, Cohen’s *d* = −0.73, a medium effect. *N* = 13 participants in each drug condition, 26 sessions. Blue and green bars represent mean performance for each drug condition; black lines represent standard error of the mean for each drug condition.

**Figure 5:**
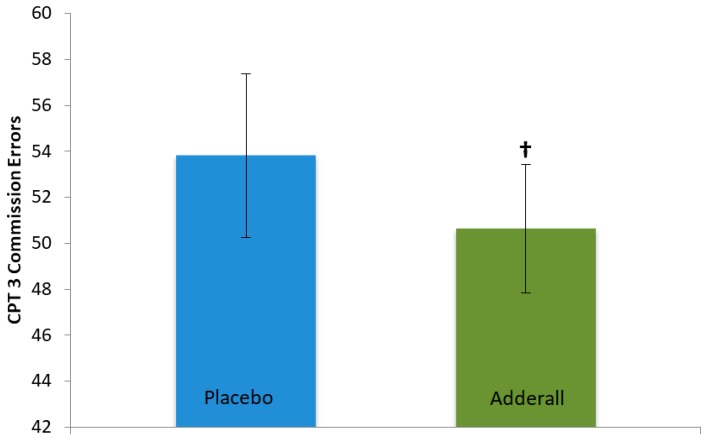
Effects of Adderall on CPT-3 Commission Errors. Legend: Adderall marginally improved performance (i.e., fewer Commission Errors): *t*(10) = −1.74, † *p* = 0.056, Cohen’s *d* = −0.30, a small effect. *N* = 13 participants in each drug condition, 26 sessions. Blue and green bars represent mean performance for each drug condition; black lines represent standard error of the mean for each drug condition.

**Figure 6:**
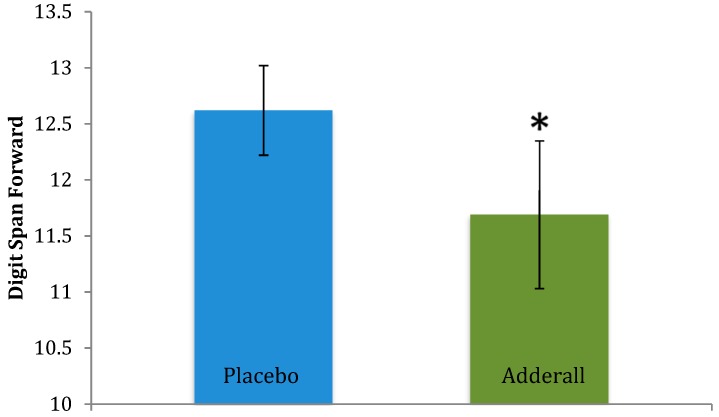
Effects of Adderall on Working Memory. Legend: Adderall worsened performance (i.e., fewer digits remembered): *t*(12) = −1.80, * *p* < 0.05, Cohen’s *d* = 0.47, a medium effect. *N* = 13 participants in each drug condition, 26 sessions. Blue and green bars represent mean performance for each drug condition; black lines represent standard error of the mean for each drug condition.

On measures of executive functioning (i.e., CPT 3) Adderall significantly reduced Variability *t*(10) = −2.25, *p* = 0.02, and marginally reduced Commission Errors, *t*(10) = −1.74, *p* = 0.056, and Hit Reaction Time *t*(10) = −1.47, *p* = 0.086 (see Table 1 and Table 3), collectively supporting that Adderall improved attention skills. CPT 3 effects are illustrated in Figure 4 and Figure 5. In contrast, Adderall *worsened* working memory on Digit Span Forward, *t*(12) = −1.80, *p* = 0.048 (Table 1 and Table 3). Working memory effects are in Figure 6. There were trend-level drug effects on BRIEF-BRI, *t*(12) = 1.54, *p* = 0.075; and BRIEF-GEC, *t*(12) = 1.35, *p* = 0.10, with a marginal worsening of participants perceptions of their past cognitive and self-regulation executive functioning in daily activities under Adderall compared to placebo. Adderall effects on the GORT and WJ were not statistically significant (*p* > 0.13 and 0.23, respectively; n.s.) suggesting that Adderall neither improved nor impaired reading performance. There were no statistically significant effects of Adderall on participants’ perceptions of whether the drug affected their own task performance, *p* = 0.30.

## 9. Discussion

A robust body of literature exists documenting widespread misuse of prescription stimulant medication on college campuses primarily for the purpose of neurocognitive and academic enhancement [9]. Much less is known, however, regarding the direct cognitive effects of prescription stimulants when taken non-medically, especially in college students without ADHD. The present, pilot study is the first to explore potential cognitive effects in conjunction with activational mood, autonomic effects and self-perceptions of drug effects on task performance in the same individuals, and provides proof-of-concept data and effect size estimates for future work. We hypothesized student performance on measures of cognition, activated emotion, subjective states and autonomic activity would be enhanced after Adderall relative to placebo, however findings indicated that Adderall lead to mixed effects including both impairment in cognitive functioning (working memory) and improvement in attention performance. These findings are generally consistent with meta-analytic findings by [29] who found small effects of amphetamine and methylphenidate on working memory and inhibitory control in healthy adults but concluded that the effects are “probably modest”. It is important to note that a robust body of literature exists that supports the positive effects of prescription stimulants on neurocognitive functioning in children and adults with ADHD (e.g., [14,54,55]), underscoring the importance of baseline impairments in performance relative to improved effects. 

With respect to autonomic activation and mood effects, our findings revealed significant drug-induced changes in activated emotion, subjective drug effects and autonomic activation (i.e., heart rate and diastolic and systolic blood pressure) that were *large* in effect size. These findings indicate that healthy college students experience substantive increases in emotional and autonomic activation in the period following Adderall consumption. These effects are consistent with the large increases in activated positive emotion, subjective drug effects, physiological activity and frontal brain glutamate in healthy young adults after consumption of other psychostimulant drugs, such as 20 mg oral d-amphetamine sulfate [36,38], 20 mg oral Desoyxn^®^ [38], and 0.6 mg/kg oral methylphenidate [37].

With regard to specific effects of Adderall on neurocognition, the results were mixed and much smaller in size. Specifically, the primary finding was that Adderall lead to reduced response variability on an executive function measure (CPT-3) relative to placebo, supporting enhanced attention skills [43]. However, it is important to note that Adderall *impaired* working memory performance relative to placebo, and was associated with a trend-level *worsening* of participants’ ratings of their own past cognitive ability and overall ability to self-regulate (BRIEF-A). These findings support that Adderall can have neurocognitive effects that are discordant with drug expectancies, and while improving attention skills, may simultaneously degrade students’ confidence in their abilities to problem solve, complete tasks, and interact with others [56]. As task-related stress is increased after stimulant drug use [57], such effects could increase student motivation to use stimulant drugs in response to academic stressors. In contrast to [29], the present study did not find evidence that participants perceived the drug had enhanced their neurocognitive performance. 

Lastly, our findings indicated that Adderall neither improved nor deleteriously affected oral reading performance or story recall suggesting that Adderall may not enhance academic performance in healthy college students although additional empirical studies are needed to explore this finding. In addition, no significant effects of Adderall on participants’ perception of a possible drug effect on cognitive performance (PDE-SR) were found, indicating that participants were largely unaware of the effects of Adderall on their own neurocognitive performance. Collectively, findings of the present suggest that Adderall does not result in robust neurocognitive enhancement benefits in healthy college students.

The strengths of the present study include the placebo-controlled, within-subjects crossover design; evaluation of a prescription psychostimulant—Adderall—that is commonly misused for cognitive enhancement by college students; time-locked procedures for drug and placebo administration; and investigation of effects in a sample of healthy young adults carefully screened for medical and psychiatric disease. Autonomic data confirmed sympathomimetic drug activity, providing evidence of internal validity of drug exposure. The small to moderate effect sizes of 30 mg Adderall on neurocognition observed here support the negligible to small effects identified at lower doses (e.g., 10 mg; [58] and indicate that Adderall effects on neurocognition are likely small in size across a broad dosing range. In contrast, Adderall had large effects on activated positive emotion, subjective drug states, and autonomic activity, indicating dissociation between the effects of Adderall on activation and neurocognition. The within-subjects design provided two times the number of data points per subject compared to a between-subjects design and entirely controlled for individual differences between the drug and placebo conditions. This design reduces error variance and maximizes statistical power compared to alternate study designs [59]. The study provides empirically determined effect size estimates to guide future work on the effects of Adderall on specific neurocognitive outcomes in healthy college students. 

Limitations of the study should also be noted. First, given the nature of the pilot study, the sample size is quite small and future studies with large samples are needed to further explore the neurocognitive effects of prescription stimulants. Studies are also needed to investigate potential placebo responding on task performance, and on perceived drug effects on task performance [11]. In addition, neurocognitive testing was conducted 90–210 min post-drug, which is the peak period of drug effects on emotion and autonomic activity. Potential drug effects on neurocognition beyond this time period were not evaluated. The study had high power to detect large effects, and lower power to detect moderate to small effects. As such the likelihood of Type II error is greater for small to medium effects. Findings with small to medium effect sizes (identified in Table 1 and Table 3) will require larger sample sizes in future work. Despite these limitations, the present study had adequate power for large effects and indicated dissociable effects of Adderall on neurocognition and activation, indicating differential acute impact on these outcomes. 

In summary, the present pilot study indicates that a moderate dose of Adderall has small to minimal effects on cognitive processes relevant to academic enhancement (i.e., on reading comprehension, fluency, cognitive functioning), in contrast with its significant, large effects on activated positive emotion, autonomic activity, and subjective drug responses. This finding supports a dissociation between effects of Adderall on activation and neurocognition. Furthermore, in the present study, Adderall improved attention performance but impaired performance on working memory performance. Such effects appear somewhat discordant with drug expectancies in healthy college students who use these drugs primarily for purposes of neurocognitive and academic enhancement. Overall, the present findings indicate that non-medical use of moderate-dose Adderall in healthy college students may improve attention ability but has minimal or adverse impact on other cognitive processes and does not enhance academic performance, despite the common misuse of stimulant drugs for these purposes. 

## Figures and Tables

**Table 1:** Neurocognitive Task Performance by Drug Condition.

Task	Placebo *M* (*SD*)	Adderall *M* (*SD*)	Cohen’s *d*
BRIEF-A—BRI ^†^	38.23 (7.47)	39.92 (7.10)	0.23
BRIEF-A—GEC ^†^	96.15 (20.10)	98.92 (19.72)	0.14
BRIEF-A—MI	57.92 (13.58)	59.00 (13.83)	0.08
CPT 3—Omission Errors	46.82 (5.23)	46.00 (4.17)	−0.17
CPT 3—Commission Errors ^†^	53.82 (11.79)	50.64 (9.23)	−0.30
CPT 3—Hit Reaction Time ^†^	49.36 (8.96)	47.46 (5.75)	−0.25
CPT 3—Variability *	45.46 (7.88)	41.00 (3.61)	−*0.73*
DS Total	34.00 (4.78)	33.46 (6.32)	−0.10
DS Forward *	12.62 (1.45)	11.69 (2.39)	−*0.47*
DS Backward	10.62 (2.63)	10.46 (2.57)	−0.06
GORT—ORI	98.77 (8.61)	99.69 (8.61)	0.11
PDE-SR	44.39 (9.55)	47.54 (20.33)	0.20
WJ	43.62 (4.86)	44.54 (5.87)	0.17

Note. BRIEF-A—BRI = Behavior Rating Inventory of Executive Function, Adult version—Behavior Regulation Index; BRIEF-A—GEC = BRIEF-A—Global Executive Composite; BRIEF-A—MI = BRIEF-A—Metacognition Index; CPT 3 = Conners’ Continuous Performance Test—Third Edition; DS = Digit Span; GORT—ORI = Gray Oral Reading Test—Oral Reading Index. PDE-SR = Perceived Drug Effect Self-Report. WJ = Woodcock-Johnson—Third Edition, Story Recall. Medium effect sizes are in *italics*. Small to negligible effect sizes are in normal font. * = significant at the 0.05 level; ^†^ = marginally significant. *N* = 13 participants in each drug condition, 26 sessions.

**Table 2:** Adderall Effects on Autonomic Activity, Subjective State and Activated Emotion.

Measure	Drug *F*(1,12)	Time *F*(10,120)	Drug × Time *F*(10,120)	Partial Eta-Squared Effect Size	Cohen’s *d* Effect Size	Direction of Effect
Heart rate	12.84 ***	3.52 ****	4.30 *****	**0.52**	**1.25**	Increased
Systolic BP	10.1 **	1.77	3.36 ****	**0.46**	**1.05**	Increased
Diastolic BP	5.38 *	1.12	3.28 ****	**0.31**	**0.86**	Increased
Feel Drug Effect	9.64 **	6.87 *****	2.82 ***	**0.45**	**1.26**	Increased
Feel High	7.06 *	3.35 ****	2.46 **	**0.37**	**1.04**	Increased
PA	5.27 *	1.18	2.20 *	**0.31**	*0.71*	Increased

Note. Increased = participant responses/reactions were greater under the effects of Adderall relative to placebo. Heart rate = beats per minute; BP = blood pressure; Feel Drug Effect = ratings on Drug Effects Questionnaire item “I feel some drug effects right now”; Feel High = ratings on Drug Effects Questionnaire item “I feel high”; PA = Morrone et al. (2000) Positive Activation; * *p* ≤0.05; ** *p* ≤0.01; *** *p* ≤0.005; **** *p* ≤0.001; ***** *p* ≤0.0001. Partial eta-squared values indicate effect size of the drug main effect across the entire test session. Cohen’s *d* values indicate effect size of the drug effect at the time point of maximal difference on the outcome. Large effect sizes are in **bold**. Medium effect sizes are in *italics*. *N* = 13 participants in each drug condition, 26 sessions.

**Table 3:** Specific Adderall Effects on Neurocognition.

Task	Placebo *M* (*SD*)	Adderall *M* (*SD*)	*t*-test	*p*	Direction of Effect
Digit Span Forward *	12.62 (1.45)	11.69 (2.39)	*−1.80*	*0.048*	*Worsened*
CPT 3—Variability *	45.46 (7.88)	41.00 (3.61)	*−2.25*	*0.024*	Improved
CPT 3—Commission Errors ^†^	53.82 (11.79)	50.64 (9.23)	−1.74	0.056	Improved
CPT 3—Hit Reaction Time ^†^	49.36 (8.96)	47.46 (5.75)	−1.47	0.086	Improved
BRIEF-A—BRI ^†^	38.23 (7.47)	39.92 (7.10)	1.54	0.075	More negative perceptions
BRIEF-A—GEC ^†^	96.15 (20.10)	98.92 (19.72)	1.35	0.10	More negative perceptions

Note: Improved = participant performance was improved under the effects of Adderall. Worsened = participant performance worsened under the effects of Adderall. More negative perceptions = participant perceptions of their executive functioning in daily activities were more negative, based on their responses to the BRIEF-A. BRIEF-A—BRI = Behavior Rating Inventory of Executive Function, Adult version—Behavior Regulation Index; BRIEF-A—GEC = BRIEF-A—Global Executive Composite; CPT 3 = Conners’ Continuous Performance Test—Third Edition; Medium effect sizes are in *italics*. Small effect sizes are in normal font. * = significant at the 0.05 level; ^†^ = marginally significant. *N* = 13 participants in each drug condition, 26 sessions.
